# Functional Characterization of Selected Universal Stress Protein from *Salvia miltiorrhiza* (SmUSP) in *Escherichia coli*

**DOI:** 10.3390/genes8090224

**Published:** 2017-09-08

**Authors:** Xiao-Fan Wang, Jiao Su, Na Yang, Hui Zhang, Xiao-Yan Cao, Jie-Fang Kang

**Affiliations:** Key Laboratory of the Ministry of Education for Medicinal Resources and Natural Pharmaceutical Chemistry, National Engineering Laboratory for Resource Development of Endangered Crude Drugs in Northwest of China, Shaanxi Normal University, Xi’an 710062, China; fanfan9138@163.com (X.-F.W.); sujiao@snnu.edu.cn (J.S.); m18829222318@163.com (N.Y.); 201503829@snnu.edu.cn (H.Z.)

**Keywords:** universal stress protein (USP), gene family, *Salvia miltiorrhiza*, *Escherichia coli*, abiotic stress

## Abstract

The multigene universal stress protein (USP) family is evolutionarily conserved. Members play indispensable roles in plant tolerance to abiotic stresses. Although relatively well-characterized in model plants, such as *Arabidopsis thaliana* and *Oryza sativa*, this family has not been investigated in *Salvia miltiorrhiza*, an important herbal plant for which yields can be limited by various abiotic stresses. Here, we identified 32 USP family members in the *S. miltiorrhiza* genome, and used phylogenetic analysis to sort these SmUSPs into four groups. Groups A and B belong to the ATP-binding class whereas Groups C and D are in the non-ATP-binding class. Motif analysis and multiple sequence alignment hinted that members of group A and B were able to bind ATP. Our qRT-PCR data from different tissues/organs and under salt and heat stresses provided an overall expression pattern for those genes. Three *SmUSP*s (*SmUSP1*, *SmUSP8*, and *SmUSP27*) were cloned from *S. miltiorrhiza* and functionally characterized in *Escherichia coli.* Compared with the control cells, those that expressed SmUSPs exhibited enhanced tolerance to salt, heat, and a combination of the two. This suggested that the protein has a protective role in cells when exposed to single-stress and multiple-stress conditions. Our findings provide valuable information that helps improve our understanding of the evolutionary and functional conservation and diversity associated with the *USP* gene family in *S. miltiorrhiza*.

## 1. Introduction

Abiotic stresses, such as drought, salinity, and extreme temperatures, are limiting factors for normal plant growth. Excessive salinity interrupts homeostasis in water potential and ion distribution at both the cellular and whole-plant level [1]. This results in molecular damage, arrested development, and even mortality [2]. High temperatures can cause irreversible damage, including protein denaturation, aggregation, and degradation [3], as well as inhibition of protein synthesis and inactivation of enzymes. Extreme temperatures can also increase the fluidity of membrane lipids, leading to a loss of membrane integrity [4]. Field environments are very different from the controlled conditions used in laboratory studies, and outdoor experiments often involve exposing plants simultaneously to more than one type of abiotic and/or biotic stress, e.g., salinity plus heat, a combination that is probably the major environmental factor that restricts growth and yields in the 21st Century [5].

The family of universal stress protein genes (*USP*s) appears to play a positive role in the abiotic-stress response, with members encoding proteins that contain highly conserved residues of the Universal Stress Protein A domain (UspA domain; Pfam Accession Number PF00582). These genes are widely distributed across most living organisms, including bacteria, archaea, fungi, protozoa, plants, and mammals [6,7,8]. The first *USP* was discovered in a bacterium, and was initially named C13.5 protein because of its migration on a two-dimensional Isoelectric Focusing-PAGE gel. That name was later changed to *USP* to reflect its ability to respond to diverse stresses. The label UspA was quickly and extensively acknowledged, and now represents an orthologous group of proteins, the UspA superfamily [9]. Six *USP* genes in *Escherichia coli* have different functions linked to motility, adhesion, and resistance to oxidative stress [10]. Homologs of *USP*s are ubiquitous in plants and encoded by gene families. They include 44 *Arabidopsis* proteins that are similar to the UspA domains in bacteria as well as 16 putative *USP* genes in barley (*Hordeum vulgare*) [6,11]. This gene family comprises two classes: ATP-binding and non-ATP-binding [12].

Although *USP* genes have been characterized in diverse plant species, including barley, *A. thaliana* (hereafter, *Arabidopsis*), *Oryza sativa*, *Gossypium arboreum*, *Astragalus sinicus*, *Vicia faba*, *Solanum pennellii*, and viridiplantae, their functions remain largely unknown [6,11,13,14,15,16,17]. It appears that *USP* improves the rate of cell survival during prolonged exposure to stress agents, and may endow plants with wide-ranging stress tolerance [18]. For example, *SpUSP* in *S. pennellii* reduces the stomatal aperture to protect plants from the effects of drought [10]. Among the *Arabidopsis USP* genes, At5g54430 and At4g27320 are phosphorylated in response to microbial elicitation of the cells [17]. At3g53990 exhibits a chaperone function and is significantly induced by heat, H_2_O_2_, and drought treatments [19]. Another *Arabidopsis USP* gene, *At3g62550*, encodes an ATP-binding motif and has been shown to be up-regulated in a drought microarray dataset [17].

*Salvia miltiorrhiza* (family Labiatae) is an important herbal plant. Its dried roots, also known as ‘Danshen’, are widely used in modern and traditional Chinese medicine (TCM) for treating cardiovascular/cerebrovascular diseases and various symptoms of inflammation [20,21,22]. This species is emerging as a model plant for TCM studies because of its relatively small genome (600 Mb), short life cycle, minimal growth requirements, and significant medicinal value [23]. Although the functions of numerous genes from *S. miltiorrhiza* have been reported, information about the *SmUSP* gene family is lacking. The genomic database of *S. miltiorrhiza* has been published online at http://www.ndctcm.org/shujukujieshao/2015-04-23/27.html [24]. Heterologous in vivo expression in *E. coli* is an approach now available for functional characterization of stress-responsive genes [25]. Here, we identified *USP* members from the genomic database of *S. miltiorrhiza*. By integrating the results from phylogenetic analysis and investigations of protein motif structures, gene expression, and heterologous expression in *E. coli*, we were able to determine the evolutionary and functional conservation and diversity of this gene family in *S. miltiorrhiza*. Our findings provide a genetic resource for improving abiotic stress tolerance by *S. miltiorrhiza* through genetic engineering technologies.

## 2. Materials and Methods

### 2.1. Identification of USP Genes from the Salvia miltiorrhiza Genomic Database

Using the conserved domain search service of the NCBI database (www.ncbi.nlm.nih.gov/Structure/cdd/wrpsb.cgi), we found 44 *Arabidopsis* protein sequences with similarity to the UspA domains of bacteria [6], and obtained 26 UspA domains. Those conserved domain sequences were used to search, with TBLASTN (e-value < 10^−10^), for the sequences of *USP*s in the *Salvia miltiorrhiza* genomic database [24]. All of the identified candidates were analyzed using the protein family database (Pfam; http://pfam.sanger.ac.uk/) to confirm the presence of UspA domains in their protein structure.

### 2.2. Bioinformatics Analysis, Phylogenetic Analysis, and Multiple Sequence Alignment

The molecular weights, theoretical pI values, and number of amino acids for the 32 SmUSPs were predicted using the Compute pI/Mw tool on the ExPASy server (http://web.expasy.org/compute_pi/) [26]. Their conserved protein motifs were examined with MEME v4.11.2 software (http://meme-suite.org/tools/meme), which revealed 10 different motifs, and minimum motif and maximum motif windows set to 6 and 50, respectively. A signal peptide analysis was conducted using the TargetP algorithm (TargetP: http://www.cbs.dtu.dk/services/TargetP/). Finally, the exon–intron structures of *SmUSPs* were determined via GSDS 2.0 (http://gsds.cbi.pku.edu.cn/), comparing the full-length cDNA sequences to the genomic sequences.

We used MEGA 6 software to construct a phylogenetic tree by the Neighbor Joining method, according to the following parameters: P-distance model, pairwise deletions, and 1000-replicates bootstrap. Multiple sequence alignments of 21 *SmUSPs* and the *USP* sequence of *Methanococcus jannaschii* MJ0577 (1MJHA; NCBI protein GI: 5107801), were generated by DNAMAN v6.0.3.99 software, with default parameters.

### 2.3. Plant Growth and Stress Treatments

For expression profiling, we placed 2-month-old, uniformly developed *S. miltiorrhiza* plants in a greenhouse (16-h photoperiod, 25 °C). Salt stress was induced by watering the plants with a 150 mM NaCl solution, heat-stress conditions were imposed by transferring the plants to a growth chamber and holding them at 38 °C. For the combined salt/heat treatment, the plants were watered with 150 mM NaCl solution, then transferred to the chamber and held at 38 °C. After 24 h, leaves were collected from different plants (three biological replicates), immediately frozen in liquid nitrogen, and stored at −80 °C. For the control, plants continued to receive normal irrigation in the greenhouse, under the conditions described above, throughout the experimental period. Their leaves were also collected and stored at −80 °C.

### 2.4. Promoter Sequence Analysis, RNA Isolation, and Quantitative RT-PCR Analysis

The 2-kb genomic sequence upstream of the 5’-UTR of each *SmUSP* was obtained for putative cis-element analysis, using the PlantCARE database (http://bioinformatics.psb.ugent.be/webtools/plantcare/html/). Quantitative RT-PCR (qRT-PCR) analysis was performed to monitor the expression levels of *SmUSPs* in the roots, stems, leaves, flowers, and seeds, as well as under various stress treatments. The gene-specific primers are listed in Table S1. Total RNA was isolated using an RNA rapid extraction kit (Xi’an Chunfeng, Xi’an, China) according to the manufacturer’s instructions. First-strand cDNA was synthesized with reverse transcriptase (Takara, Beijing, China) by following the manufacturer’s protocol, and qRT-PCR was conducted on a LightCycler 96 system (Roche, Basel, Switzerland). The reaction mixture contained 10 μL of 2 × SYBR Premix Ex Taq II (Takara, Beijing, China), 500 nM each of sense and antisense primers, 20 ng of first-strand cDNA. Initial thermal-cycling at 95 °C for 30 s was followed by 45 cycles of 95 °C for 10 s and 60 °C for 30 s. Ubiquitin (GenBank Accession No. JF760206.1) served as the internal control. Relative gene expression levels were analyzed according to the comparative 2^−ΔΔC(T)^ method [27].

### 2.5. Molecular Cloning and Construction of Escherichia coli Strains Expressing Universal Stress Proteins

Full-length opening read frames of *SmUSP*1, *SmUSP*8, and *SmUSP*27 were cloned using high-fidelity ARHS DNA polymerase (Takara), and restriction sites were added to the primers (Table S2). The PCR conditions consisted of an initial denaturation at 98 °C for 1 min; then 30 cycles at 98 °C for 10 s, 55 °C/58 °C/59 °C for 25 s, and 72 °C for 60 s; followed by a final extension at 72 °C for 10 min. The PCR products were digested with SacI and XhoI/BamHI and HindIII/BamHI and XhoI (New England BioLabs, or NEB, Ipswich, MA, USA). Afterward, the fragments of *SmUSP*s, with sticky SacI, XhoI/BamHI, HindIII/BamHI, and XhoI (NEB), were inserted into pET28a vectors and sequenced to create recombinant plasmids pET28a–*SmUSP*s.

These plasmids plus the empty vector pET28a were transformed into *E. coli* BL21 strain. Cells carrying either pET28a–*USP*s or pET28a were cultured overnight at 37 °C in Luria-Bertani (LB) liquid media containing 50 mg L^−1^ kanamycin (kan). They were then diluted 1000 times with fresh LB liquid media containing 50 mg L^−1^ kan and incubated continually until the cells reached the mid-log phase (OD_600nm_ = 0.6). Isopropyl b-d-thiogalactopyranoside (IPTG) was added to a final concentration of 1 mM and the cells were cultured for another 6 h. They were then harvested by centrifugation, re-suspended in LB liquid media, and mixed with 5 × SDS loading buffer (Zhuangmeng Biology, Beijing, China). After being held in a 100 °C water bath for 5 min, the mixture was used for SDS-PAGE analysis. For western blot, HIS-Tag mouse monoclonal antibody was used for detection (Beijing Biodragon Immunotechnologies Co., Ltd., Beijing, China) along with HRP Goat anti-mouse Ig (H + L) (Abbkine Scientific Co., Ltd., California, USA). The immunoreactive bands were detected by chemiluminescence and pictured with the ChemiDoc™ MP Imaging System (BIO-RAD, Hercules, CA, USA).

### 2.6. Salt, Heat, and Combined Treatments with Transformed E. coli Cells

Cell cultures of *E. coli* were grown as described above. After IPTG induction for 6 h, the OD_600_ was determined for each sample so that they could be equilibrated to the same value. Afterward, each sample was diluted 10, 100, or 1000 times. For studying their tolerance to salinity, 7-μL samples from those different dilutions were spotted onto LB solid media containing 50 mg L^−1^ kan plus 200 mM NaCl. The plates were incubated upside-down overnight at 37 °C. Samples with the same OD_600_ value were then inoculated into an LB liquid medium (1:200 ratio) containing 50 mg L^−1^ kan plus 200 mM NaCl and measurements were made at 2-h intervals to produce a growth curve.

For evaluating their degree of heat tolerance, samples of the different dilutions were exposed to 50 °C for 60 min before 7-μL aliquots were spotted on LB media containing 50 mg L^−1^ kan. The plates were incubated upside-down overnight at 37 °C. Samples with the same OD_600_ value were exposed to 50 °C for 30 min before being inoculated into an LB liquid medium (1:200 ratio) containing 50 mg L^−1^ kan. Measurements were made at 2-h intervals to produce a growth curve.

For studying their tolerance to the combination of salt and heat stressors, the same procedures as for evaluating heat tolerance were utilized, except that the LB medium contained both 50 mg L^−1^ kan and 200 mM NaCl.

### 2.7. Statistical Analysis

All data were presented as the means ± Standard Deviation (SD) of at least three replicates. Statistical analysis was performed with SPSS 17.0 software (IBM, Armonk, NY, USA). Analysis of variance (ANOVA) was followed by Tukey’s pairwise comparison tests. Mean values were considered significantly different at *p* < 0.05.

## 3. Results

### 3.1. Gene Identification, Features of Sequences, and Phylogenetic Analysis

We identified 32 *SmUSP* genes that contain the UspA domain. They were named *SmUSP1* through *SmUSP32*, based on their annotated gene IDs (NCBI Accession Number: MF614040 to MF614071). *Salvia miltiorrhiza* has fewer *USP*s than *Arabidopsis*. The genomic DNA of the former group ranges in length from 362 bp (*SmUSP11*) to 5478 bp (*SmUSP5*), while the cDNAs are 279 bp (*SmUSP11*) to 2274 bp (*SmUSP3*) long (Table S3). The molecular weights of the predicted SmUSPs vary from 10,361.16 Da (SmUSP11) to 83,958.85 Da (SmUSP3), while the theoretical isoelectric points are predicted to range from 4.87 (SmUSP15) to 11.16 (SmUSP30).

**Figure 1:**
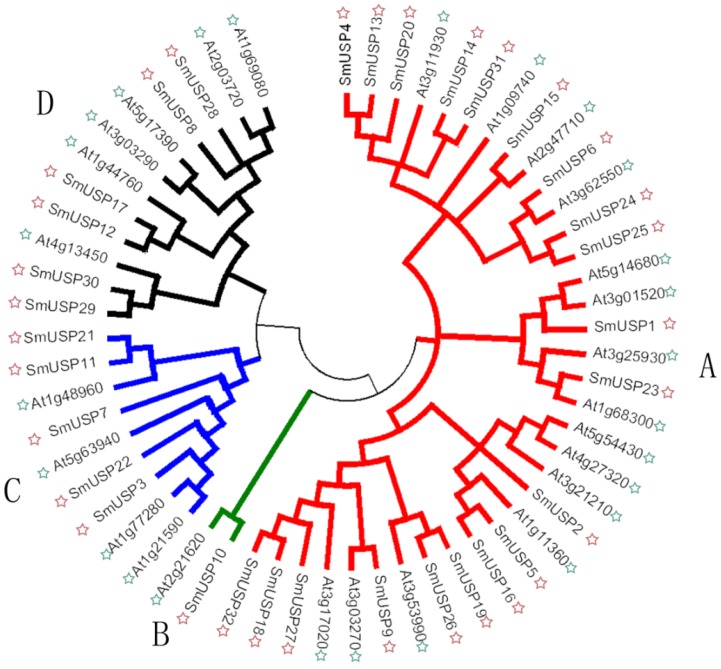
Phylogenetic relationship of *Salvia miltiorrhiza* and *Arabidopsis thaliana* universal stress protein genes (*USPs*) that contain UspA domain. *USP* groups are distinguished by color of branch. Green stars, *USPs* from *Arabidopsis*; red stars, *USPs* from *S. miltiorrhiza*.

A phylogenetic analysis based on full-length protein sequences revealed that these 32 SmUSPs can be arranged into four groups together with the 26 *Arabidopsis* USPs that also contain the UspA domain, thereby confirming that all belong to the same gene family (Figure 1). Subfamily A has the most, i.e., 20 SmUSPs while subfamily B has only one member, SmUSP10.

### 3.2. Conserved Motifs, Multiple Sequence Alignments, Genetic Structures, and Analysis of Subcellular Locations

**Figure 2:**
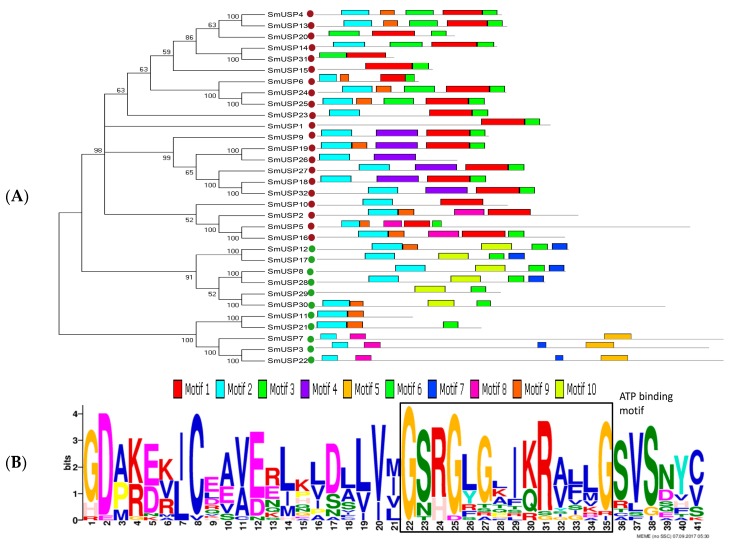
Conserved motifs of SmUSP. (**A**) Conserved motifs and phylogenetic tree of 32 SmUSPs. Red spots, members of ATP-binding class; green spots, members of non-ATP-binding class; (**B**) ATP-binding motif (G-2X-G-9X-G[S/T]) is marked.

We identified 10 motifs that contain 6 to 50 residues (Figure 2A and Figure S1). The motif patterns are conserved among the 32 SmUSPs and members within each group have similar organizations. For example, Motif 2 is shared by 27 SmUSPs; Motif 3, by 24 SmUSPs. Motif 1 contains the ATP binding motif (G-2X-G-9X-G[S/T]), which is distributed only in subfamilies A and B (Figure 2B) [28]; Motif 10 is found only in subfamily C; Motif 4 is shared by six members (SmUSP9, 18, 19, 26, 27, and 32) in subfamily A; Motif 6 is shared by seven members (SmUSP4, 13, 14, 20, 24, 25, and 31) in subfamily A; and Motif 5 is shared by three members (SmUSP3, 7, and 22) in subfamily C.

**Figure 3:**
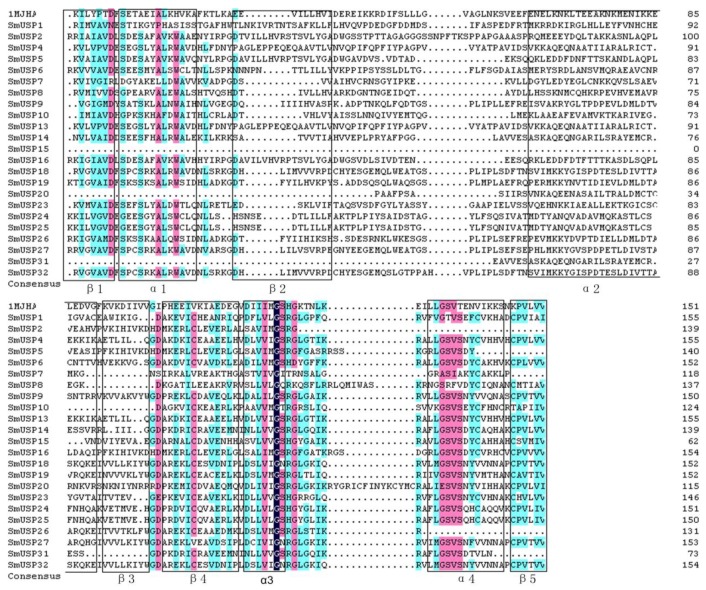
Multiple sequence alignment of 21 SmUSPs and USP MJ0577 (1MJH). The secondary structures involved in ATP-binding function of SmUSPs are marked below sequences according to structure of MJ0577 (1MJH).

Motif 1 is distributed only in subfamilies A and B, which indicates that all 21 members have the ATP-binding structure. To confirm the sequence similarity of those 21 SmUSPs to the USP sequence of *M. jannaschii* MJ0577 (1MJHA), we performed a multiple sequence alignment (Figure 3).

**Figure 4:**
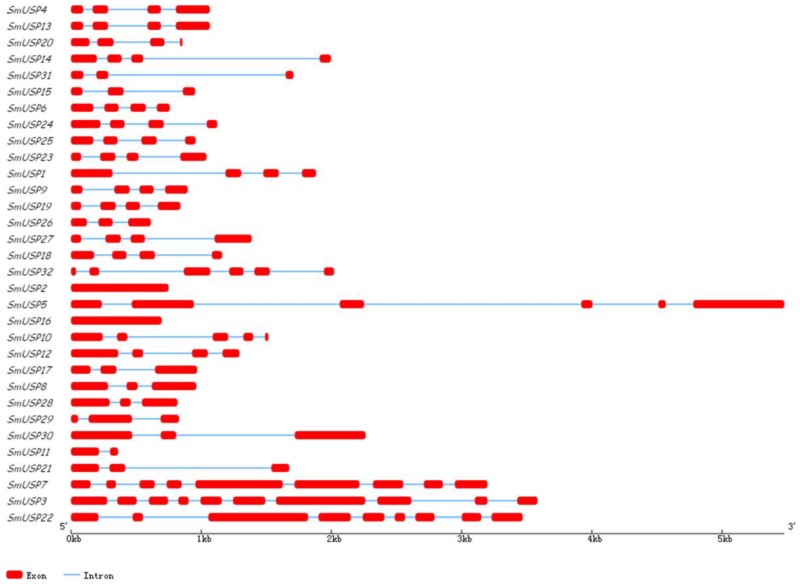
Exon–intron organization structures of 32 *SmUSP*s. Exons are represented by red, round-cornered rectangles; blue lines connecting 2 exons represent introns. Numbers above lines indicate intron phases.

Analysis of the exon–intron organization structures of all *SmUSP*s revealed that they contain one to 10 exons, while two genes (in subfamily A) lack introns (Figure 4). An excess of symmetric exons and phase-0 introns is likely to facilitate exon-shuffling, recombinational fusion, and an exchange of protein domains [29,30]. Among the 100 introns found in these *SmUSP*s, 40 are phase 0, 28 are phase 1, and 32 are phase 2. Our analysis of the *SmUSP* structures demonstrated that these genes are relatively well-conserved.

We then predicted subcellular distributions and found that the 21 SmUSPs are localized in the cytosol or nucleus (in Table S3, “other” means that no signal peptide was detected). Our results showed that SmUSP8, SmUSP15, SmUSP27, and SmUSP28 are probably targeted to mitochondria; SmUSP2, SmUSP5, SmUSP20, and SmUSP29 are probably targeted to chloroplasts; and SmUSP2 and SmUSP16 are exclusively targeted to a secretory pathway.

### 3.3. Patterns of Expression for SmUSP Genes in Various Tissues/Organs

**Figure 5:**
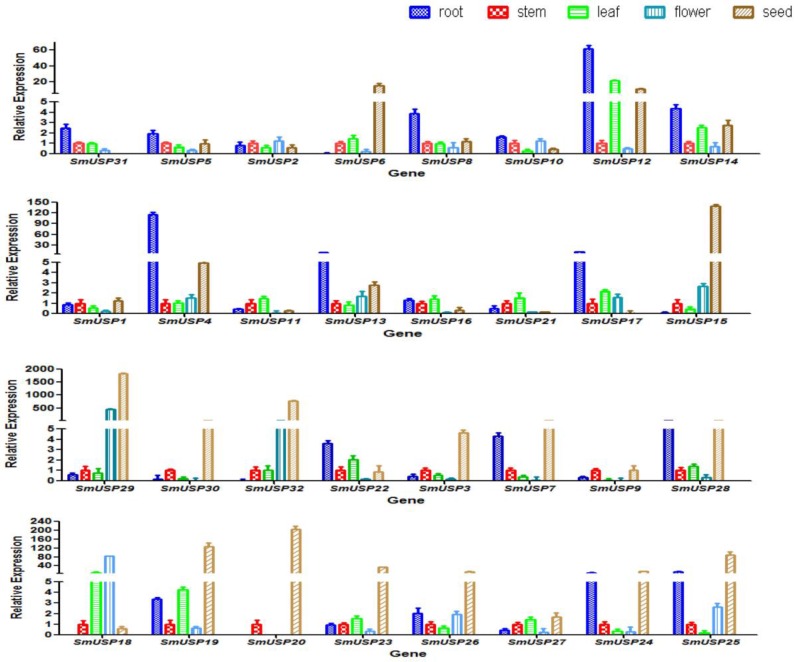
Expression patterns of 32 *SmUSP* genes in different tissues/organs. Transcription levels were analyzed according to comparative 2^−ΔΔC(T)^ method; all expression levels are relative to that detected in stem samples because all 32 *SmUSPs* expressed in stem. All data were presented as the means ± Standard Deviation (SD) of at least three replicates.

To profile the expression patterns of *SmUSP* genes in *S. miltiorrhiza*, we sampled different tissues and organs for analysis by qRT-PCR (Figure 5). In all, 29 genes were expressed in all five plant parts, while some exhibited diverse profiles. For example, expression was highest in the seeds for 16 *SmUSP*s and in the roots for 10 genes. Moreover, *SmUSP11*, *SmUSP16*, and *SmUSP21* showed the highest expression in leaves; *SmUSP2* and *SmUSP18*, in flowers; and *SmUSP9*, in stems. While *SmUSP31* was not expressed in the seeds, nor *SmUSP18* in the roots, *SmUSP20* was specifically expressed in both stems and seeds.

### 3.4. Analysis of Promoter Sequences and Differential Expression of SmUSP Genes in Response to Salt and Heat Stresses and Their Combination

We also investigated the putative stress-responsive cis-elements within the regions of 2-kb genomic sequences upstream of the 5’-UTR of these 32 *SmUSP*s (Table S4). Among them, 18 contained the ABRE responsible for responses to salinity, dehydration, heat, and cold; 20 had the HSE element involved in heat stress-responsiveness; and 22 carried the MBS element that is a transcription factor MYB binding site involved in drought-inducibility.

**Figure 6:**
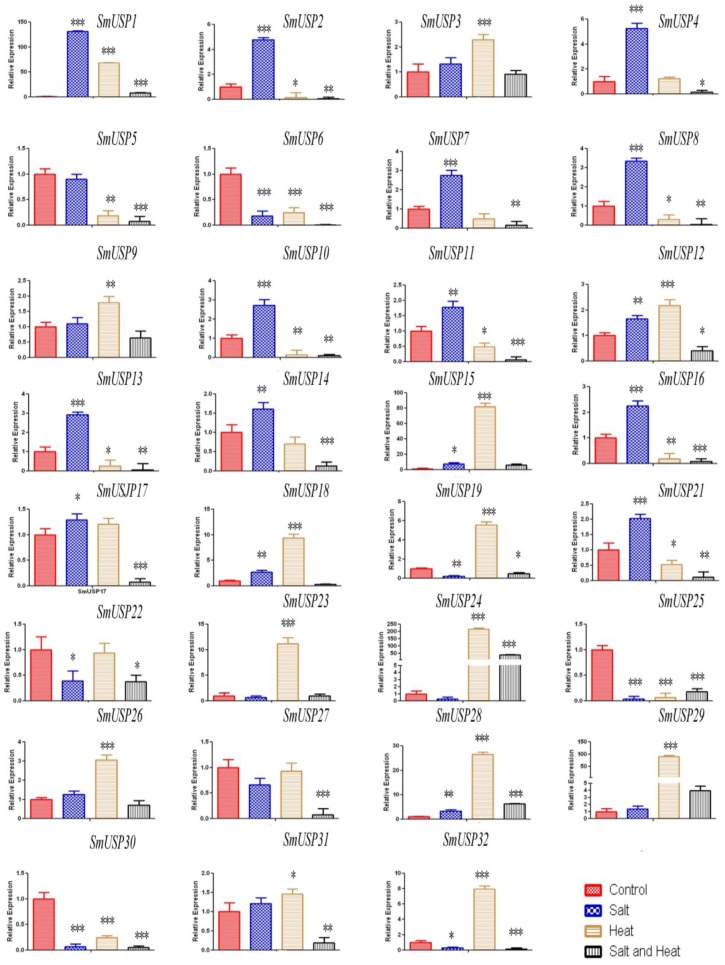
Expression of 32 *SmUSP* genes under different stress conditions. Based on comparative 2^−ΔΔC(T)^ method, all levels are relative to control. For each gene, *, **, and *** indicate that expression is significantly different from control at *p* < 0.5, 0.1, and 0.01, respectively. All data were presented as the means ± Standard Deviation (SD) of at least three replicates.

Expression pattern of different *SmUSP* genes were shown in response to either single or combinatorial stress (Figure 6). Our qRT-PCR result showed that 14 *SmUSPs* (*SmUSP1*, *2*, *4*, *7*, *8*, *10*, *11*, *12*, *13*, *14*, *16*, *17*, *18*, *21*) were significantly induced after salt stress, while 8 (*SmUPS6*, *15*, *19*, *22*, *25*, *28*, *30*, *32*) were significantly repressed. For the heat treatment, 13 *SmUSPs* (*SmUSP3*, *9*, *12*, *15*, *18*, *19*, *23*, *24*, *26*, *27*, *29*, *31*, *32*) were significantly upregulated, while 11 (*SmUSP2*, *5*, *6*, *8*, *10*, *11*, *13*, *16*, *21*, *25*, *30*) were significantly downregulated. After the salt/heat combination treatment, most *SmUSP* genes were significantly downregulated, while *SmUSP24*, *28* were exclusively upregulated.

### 3.5. Heterologous Expression and Functional Validation in E. coli

**Figure 7:**
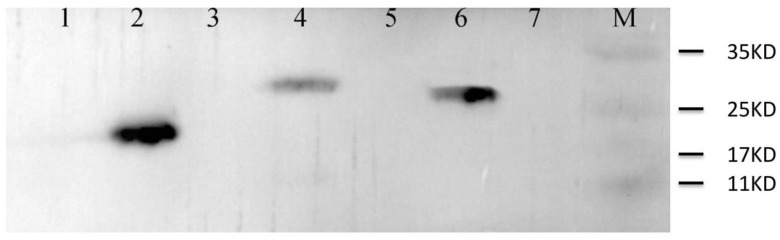
Western blot analysis of SmUSP-overexpression in *E. coli* BL21. Lanes 1, 3, 5: *E. coli* BL21 strain harboring pET28a-*SmUSP27*, pET28a-*SmUSP8*, and pET28a-*SmUSP1* without induction, respectively. Lanes 2, 4, 6: *E. coli* BL21 strain harboring pET28a-*SmUSP27*, pET28a-*SmUSP8*, and pET28a-*SmUSP1* induced for 6 h, respectively. Lane 7: *E. coli BL21* strain harboring empty pET28a induced for 6 h. M: marker.

Transformation of pET28a–*SmUSP1*, pET28a–*SmUSP8*, and pET28a–*SmUSP27* into *E. coli* BL21 produced protein profiles showing overexpression of approximately 27 kDa, 30 kDa, and 24 kDa recombinant protein, respectively, which was approximately 3 kDa larger than their predicted values (Figure 7).

**Figure 8:**
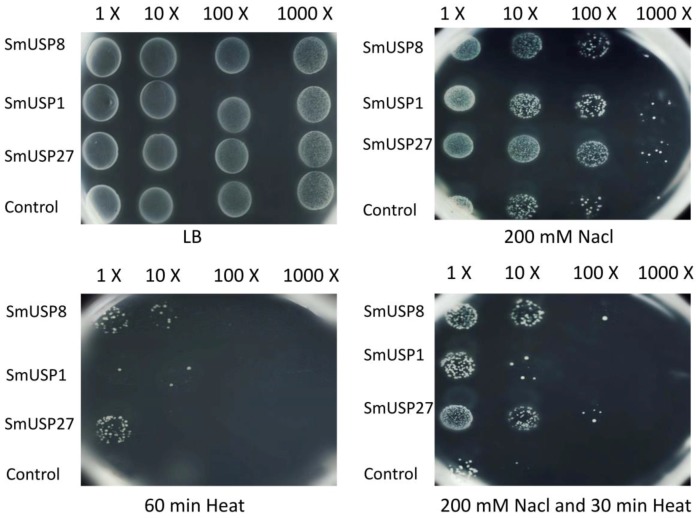
Overexpession of *SmUSP1*, *8*, *27* in *E. coli* enhanced the tolerance to different stresses by spot assay. Each sample was diluted 10-fold, 100-fold, and 1000-fold and spotted onto Luria-Bertani (LB) plates to assess tolerance to various stress treatments. LB represents *E. coli* BL21 strains transformed plasmids pET28a–*SmUSPs* and the empty vector pET28a were spotted onto LB solid media; 200 mM NaCl represents those strains were spotted onto LB solid media containing 200 mM NaCl; 60 min Heat represents those strains were exposed to 50 °C for 60 min before 7-μL aliquots were spotted on LB media; 200 mM NaCl and 30 min Heat represents those strains exposed to 50 °C for 30 min before 7-μL aliquots were spotted on LB media containing 200 mM NaCl.

The effect of *SmUSP* expression on stress tolerance was studied with *E. coli* cells. In the spot assay, the number of bacterial colonies expressing each of three genes was compared with that of the control (empty-vector) cells (Figure 8). Under salt, heat, or combined-stress treatment, more colonies containing the constructs expressed *SmUSP1*, *SmUSP8*, or *SmUSP27* than did the control, indicating that all of these selected genes were able to enhance stress tolerance in that organism. In particular, cells expressing *SmUSP27* showed greater tolerance to salt and to the combination treatment when compared with cells expressing *SmUSP1* or *SmUSP8*. In contrast, the level of tolerance to heat stress was strongest in cells expressing *SmUSP8*.

**Figure 9:**
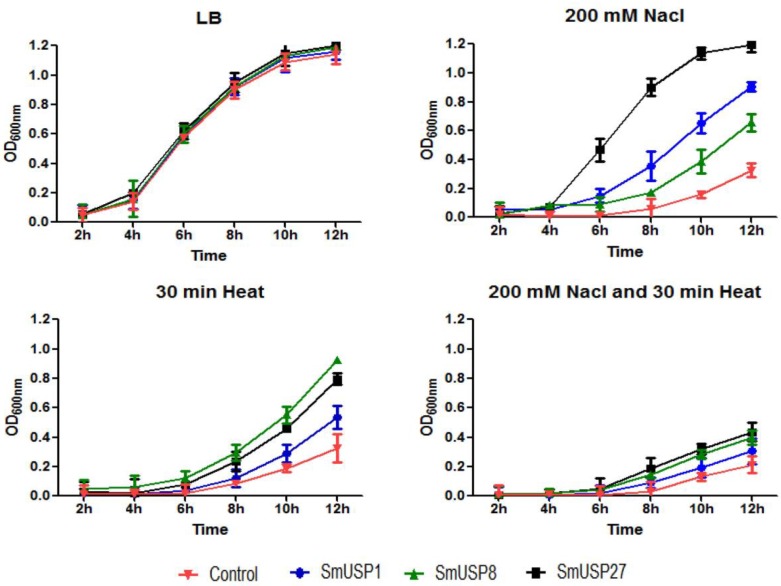
Overexpession of *SmUSP1*, *8*, *27* in *E. coli* enhanced the tolerance to different stresses by liquid assay. Each sample was diluted 200-fold, using fresh LB media, and absorbance (OD_600nm_) was measured for 12 h at 2-h intervals. Each sample has three biological replicates. LB represents samples with the same OD_600_ value were inoculated into an LB liquid medium (1:200 ratio); 200 mM NaCl represents those samples were inoculated into an LB liquid medium (1:200 ratio) containing 200 mM NaCl; 30 min Heat represents those samples were exposed to 50 °C for 30 min before being inoculated into an LB liquid medium (1:200 ratio); 200 mM NaCl and 30 min Heat represents those samples were exposed to 50 °C for 30 min before being inoculated into an LB liquid medium (1:200 ratio) containing 200 mM NaCl.

For the liquid culture assay, we measured absorbance (OD_600nm_), which reflected the extent of bacterial growth (Figure 9). Under stress conditions, the activity of *E. coli* cells expressing SmUSP genes increased significantly over time when compared with the control. Salt treatment caused absorbance values for cells harboring *SmUSP1*, *SmUSP8*, and *SmUSP27* to rise by 2.8-, 2.0-, and 3.7-fold, respectively, over control levels when cells were cultured for 12 h. After heat treatment was applied, absorbance was 1.9-, 3.2-, and 2.8-fold higher, respectively, than in the control samples, while those increases were 1.4-, 1.9-, and 2.0-fold higher, respectively, than those calculated for the control when cells were exposed to the combined salt/heat treatment. Therefore, the resulting trends were consistent between the spot and liquid culture assays.

## 4. Discussion

### 4.1. Members of the SmUSP Gene Family and Their Evolution

Our study is the first to report the identification and characterization of USPs based on the entire genome sequence of *S. miltiorrhiza*. We identified 32 universal stress proteins in *S. miltiorrhiza*. A search of the NCBI database showed that *SmUSP3*, *7*, and *22* (belonging to subfamily D) contain both an UspA domain and a PKinase domain, while the others have only the UspA domain, similar to what is found with *A. thaliana* [6]. Our phylogenetic analysis led us to organize the 32 *SmUSP*s into four subfamilies, A through D. The two UspA classes of bacteria include one that binds ATP, i.e., *M. jannaschii* MJ0577 (1MJH) and one that does not, i.e., *Haemophilus influenzae* universal stress protein (1JMV) [6]. Further analysis revealed that Motif 1 in *Salvia* contains the ATP-binding domain and appears in subfamilies A and B, where members belong to the ATP-binding class. The other SmUSPs belong to the non-ATP-binding class. Based on their exon–intron organizational structures, these 32 SmUSP genes are relatively well-conserved. Results from those examinations plus the phylogenetic analysis and multiple sequence alignment suggest that the family members have a common ancestor and have diverged over time.

### 4.2. Variations in Gene Expression Patterns

In *S. miltiorrhiza*, most of the *SmUSP*s displayed ubiquitous but highly variable expression in all tissues/organs studied, which suggested functional divergence. Because the 29 genes were detected in all plant parts, it demonstrates that members of this gene family are widely distributed and expressed in that species, and are undoubtedly involved in normal plant growth and development. They include 16 *SmUSP*s that showed the highest expression in seeds. An *Arabidopsis USP*, *At3g53990* (subfamily A), was widely expressed in most tissues, including the root, stem, leaf, and flower, and was most strongly expressed in the stem. This *Arabidopsis USP* exhibits a chaperone function, and is induced significantly by heat, H_2_O_2_, and drought [19]. *SmUSP9*, similar in sequence to *At3g53990*, showed the highest expression in stems and was induced significantly by heat. Another *Arabidopsis* USP, *At3g62550* (subfamily A), encodes an ATP-binding motif and is up-regulated in a drought microarray dataset [17]. An *SpUSP* gene from *Solanum pennellii* is highly expressed in leaves and stomata, and can reduce the stomatal aperture to protect plants from drought [10]. Both *SmUSP11* and *SmUSP21* (100% bootstrap values between them; Figure 1) showed the highest expression in leaves. Their promoter sequences contain a *cis*-element involved in circadian control, similar to one found in an *SpUSP* gene (Table S4). These results imply that *SmUSP11* and *SmUSP21* can reduce stomatal apertures to protect plants of *S. miltiorrhiza* against the effects of drought; both were induced significantly by heat and drought. *SmUSP10*, with high sequence similarity to *SLRd2*, both of which are strongly expressed in roots, might enable *S. miltiorrhiza* to resist oxidative stress [31]. Thus, our findings that most of these genes are induced by salt, heat, or a combination of the two, is consistent with the results from our analysis of their promoter sequences (Figure 6 and Table S4).

### 4.3. SmUSP Genes Enhance Stress Tolerance in E. coli

The heterologous expression of a *USP* gene from *Salicornia brachiata* in *E. coli* enhances tolerance to salt and osmotic stresses [25]. We selected *SmUSP1*, *SmUSP8*, and *SmUSP27* because of their heterologous expression in *E. coli*. All three were expressed in the five tissue/organ samples, with *SmUSP1* and *SmUSP27* belonging to the ATP-binding class and *SmUSP8* belonging to the non-ATP-binding class. Our results showed that, although *SmUSP1*/*8*/*27* responded differentially to stress conditions, their related proteins conferred tolerance to salt, heat, and the combination treatments when applied to *E. coli*. Compared to *SmUSP8*, *SmUSP27* and *SmUSP1* conferred greater tolerance to salinity in that organism while *SmUSP8* conferred the highest tolerance to heat stress. This was probably because the two separate classes of *USP* exhibit different functions in *S. miltiorrhiza.* For example, whereas *SmUSP27* induced the highest tolerance against both salt stress and the combination treatment in *E. coli*, transcription profiling indicated that *SmUSP8* was significantly up-regulated only by heat stress. Results from previous studies have also demonstrated that the response of a plant to a combination of two or more stress conditions is unique and one cannot directly extrapolate this to the response a plant might have to those stresses when applied individually [5].

### 4.4. Function of SmUSPs under Simultaneous Stresses

In contrast to having controlled growth conditions when experiments are conducted in the laboratory, a field environment often involves the simultaneous exposure of plants to more than one type of abiotic and/or biotic stress [5]. Our results indicated that the three selected *Sm*USPs are less tolerant to a combined stress than to salt or heat stress alone. Among the 32 *SmUSP* genes, 24 were significantly induced by the combined treatment while 22 and 25 were significantly induced by salt and heat stress, respectively (Figure 6). This led to a high degree of complexity in plant responses that are largely controlled by different, and sometimes opposing, signaling pathways that may interact and inhibit each other [4]. For example, in a study of *Triticum aestivum*, researchers found that the combined effects of drought plus heat on grain yields were greater than the influence that those stressors had individually [32]. In contrast, tomato plants were much better protected against the combination of salt and heat stresses than they were when challenged with salinity alone [33]. Our data demonstrate how important it is that we develop new plant lines with enhanced tolerance to various stress combinations. With that goal, we propose that future investigations into the functions of all SmUSPs should also be validated in both *E. coli* and *S. miltiorrhiza*.

## Supplementary Materials

The following are available online at www.mdpi.com/2073-4425/8/9/224/s1, Figure S1: Motif 2-10 of SmUSP proteins, Table S1: Primers for qRT-PCR, Table S2: Primers for cloning the full-length of 3 *SmUSPs*, Table S3: The characteristics of the 32 SmUSP proteins, Table S4: Promoter sequences analysis of 32 SmUSPs.
