# Supplementary material for: Functional Characterization of Selected Universal Stress Protein from Salvia miltiorrhiza (SmUSP) in Escherichia coli
Source: Genes (Basel). 2017 Sep 8;8(9):224. doi: 10.3390/genes8090224 (PMC5615357; doi:10.3390/genes8090224)
Supplement: Supplementary file 1 [file genes-08-00224-s001.zip › Table S1.pdf]

Table S1: Primers for qRT-PCR

Gene	Primer name	Sequence(5’-3’)
SmUSP1	*SmUSP1-S*	TTCCATAAGCAGCACTGGAG
	SmUSP1-A	CAGGGACTTGAACGTGAAGA
SmUSP2	*SmUSP2-S*	ATGGAGGAGGAGTACGACCA
	SmUSP2-A	CCTTCACGATGTGGATCTTC
SmUSP3	*SmUSP3-S*	CACAATCCAATCTGCAATCC
	SmUSP3-A	CTTTGCTCGATGCTGCTTAG
SmUSP4	*SmUSP4-S*	CTGTGAAGAAAGCGCAAGAG
	SmUSP4-A	CAACATGCAGTTCCTCTGCT
SmUSP5	*SmUSP5-S*	AGCAGAAACTGGAGGACGAT
	SmUSP5-A	AAGCACAGCCTCTCCTTCAT
SmUSP6	*SmUSP6-S*	GCAGTGGACGAAAGTGAAGA
	SmUSP6-A	AAGAAGGAATGGGTGGTTTG
SmUSP7	*SmUSP7-S*	AGCTGCCTAAGGATTTGGAA
	SmUSP7-A	CAGCCTCCTCTTCCGATAAG
SmUSP8	*SmUSP8-S*	AAAGACCTGAGGTGCATGTG
	SmUSP8-A	ATGATTGCTTTCGTTGTCCA
SmUSP9	*SmUSP9-S*	CCCTGATCCAGAGGTTCTTG
	SmUSP9-A	TCACACAATTTCTCCCTTGG
SmUSP10	*SmUSP10-S*	ACATCATGATCGCTGTGGAT
	SmUSP10-A	TCAAACTGGAGATGGCGTAG
SmUSP11	*SmUSP11-S*	GATAACAGCCCTCAAATGGG
	SmUSP11-A	CGGAGCTTCTTCTTGCTCTT
SmUSP12	*SmUSP12-S*	CACCCACGTCACTAACAAGG
	SmUSP12-A	GACGTGGCAAGGTAGTGAGA
SmUSP13	*SmUSP13-S*	CTGTGAAGAAAGCGCAAGAG
	SmUSP13-A	CAACATGCAGTTCCTCTGCT
SmUSP14	*SmUSP14-S*	GGCAGTCGAAGAGATGAACA
	SmUSP14-A	TTTCTTGGATGGTTGCTTGA
SmUSP15	*SmUSP15-S*	GTTGGGTCTCTGTGGGATCT
	SmUSP15-A	ACCGACGACTAGGACAGAGG
SmUSP16	*SmUSP16-S*	TCGATACGTTCACCACCACT
	SmUSP16-A	GAGCCGCTCCTTCATATCAT
SmUSP17	*SmUSP17-S*	CCTCTTCTCACCTTGCTTCC
	SmUSP17-A	ATCTTTGGTCCTTGAATGGC
SmUSP18	*SmUSP18-S*	TGAGCGATTTCACAAACTCC
	SmUSP18-A	ATGCAGTGGTGACGATGTCT
SmUSP19	*SmUSP19-S*	TGGCTGATAAAGGCGATACA
	SmUSP19-A	ATCAGAGGAGAACCGGATTG
SmUSP20	*SmUSP20-S*	AGGAGGGATCCAAAGGAAAT
	SmUSP20-A	GAGCCCACGGCTACCTATAA
SmUSP21	*SmUSP21-S*	GTGGCGATAACAGCTCTCAA
	SmUSP21-A	CGGAGCTTCTTCTTGCTCTT

SmUSP22	*SmUSP22-S*	ATTTGGTTGGGACATGAGGT
	SmUSP22-A	AATCCGACAACTGTGGTTCA
SmUSP23	*SmUSP23-S*	TCCAAGCTCGTCATCTTCAC
	SmUSP23-A	CTCCAACAAAGCTTCAGCAA
SmUSP24	*SmUSP24-S*	GGTCAAGAAAAGGAGAGTTTCC
	SmUSP24-A	TCTCCTCGCCTTCATCAA
SmUSP25	*SmUSP25-S*	ATGGGAGAAAAGGGGAAGAA
	SmUSP25-A	AGCAAATTTTGGAGGCACC
SmUSP26	*SmUSP26-S*	CGGTTCTCCTCTGATTCCAT
	SmUSP26-A	CTTTCTGCCTAGCTGCAGTG
SmUSP27	*SmUSP27-S*	AAGCTTTGAAGTGGGCTGTT
	SmUSP27-A	GAACTCGCTCAATGGAATCA
SmUSP28	*SmUSP28-S*	TGGTGGTTCTGCTCTATGTCA
	SmUSP28-A	CTTCCACCACCTCAACCTCT
SmUSP29	*SmUSP29-S*	TCATTCAAAGGACTGGCAGA
	SmUSP29-A	TCCTCCGTTAGCCTTTCTCT
SmUSP30	*SmUSP30-S*	CCGGAAGTCAAAGGAGGTAG
	SmUSP30-A	CGAGCCCTAATTTCAGTCGT
SmUSP31	*SmUSP31-S*	GGCAGTCGAAGAGATGAACA
	SmUSP31-A	TGAGCACAGTATCGCTCACA
SmUSP32	*SmUSP32-S*	TGAGCGATTTCACAAACTCC
	SmUSP32-A	ATGCAGTGGTGACGATGTCT
