# Supplementary material for: Functional Characterization of Selected Universal Stress Protein from Salvia miltiorrhiza (SmUSP) in Escherichia coli
Source: Genes (Basel). 2017 Sep 8;8(9):224. doi: 10.3390/genes8090224 (PMC5615357; doi:10.3390/genes8090224)
Supplement: Supplementary file 1 [file genes-08-00224-s001.zip › Table S2.pdf]

Table S2: Primers for cloning the full-length of 3 *SmUSPs*

Gene	Primer name	Sequence(5’-3’)
SmUSP1	*SmUSP1-F*	**GCCGAGCTC**ATGGCAGCAGCGGAGG
	SmUSP1-R	**CCGCTCGAG**TCAGTCATCAACAGGATCCTGA
SmUSP8	*SmUSP8-F*	**CGCGGATCC**ATGCCTGGTTTCTGCATGAGT
	SmUSP8-R	**CCCAAGCTT**CTAGGCCAGAAGCCAAAAGTT
SmUSP27	*SmUSP27-F*	**CGCGGATCC**ATGGCAGATTGCAGAAGAGT
	SmUSP27-R	**CCGCTCGAG**CTAGTCGTGGCTCTTCACTACAG
