# Supplementary material for: Functional Characterization of Selected Universal Stress Protein from Salvia miltiorrhiza (SmUSP) in Escherichia coli
Source: Genes (Basel). 2017 Sep 8;8(9):224. doi: 10.3390/genes8090224 (PMC5615357; doi:10.3390/genes8090224)
Supplement: Supplementary file 1 [file genes-08-00224-s001.zip › Table S3.pdf]

Table S3: The characteristics of the 32 SmUSP proteins

Gene	Gene length （bp）	CDs length (bp)	Protein(aa)	Mw（Da）	P*I*	Subcellular location
SmUSP1	1882	669	222	24705.22	6.81	other
SmUSP2	750	750	249	26513.96	10.69	chloroplast
SmUSP3	3583	2274	757	83958.85	6.08	other
SmUSP4	1067	579	192	20958.36	6.08	other
SmUSP5	5478	1749	582	64848.89	5.49	chloroplast
SmUSP6	759	501	166	18356.15	6.81	other
SmUSP7	3199	2265	754	83109.59	5.20	other
SmUSP8	963	714	237	27260.38	10.20	mitochondrion
SmUSP9	897	492	163	17683.62	9.27	other
SmUSP10	1516	546	181	20173.33	5.78	other
SmUSP11	362	279	92	10361.16	9.30	secretory pathway
SmUSP12	1294	720	239	26430.74	9.14	other
SmUSP13	1067	579	129	20958.36	6.08	other
SmUSP14	1997	489	162	17546.36	9.16	other
SmUSP15	954	306	101	10740.33	7.76	mitochondrion
SmUSP16	711	711	236	25679.81	4.87	chloroplast
SmUSP17	969	597	198	21795.30	9.36	other
SmUSP18	1161	486	161	17723.45	6.72	other
SmUSP19	841	483	160	18005.87	6.29	other
SmUSP20	856	399	132	14830.45	9.73	chloroplast
SmUSP21	1677	477	158	17648.63	9.61	secretory pathway
SmUSP22	3469	2091	696	76039.63	6.00	other
SmUSP23	1041	492	163	18121.86	7.07	other
SmUSP24	1124	540	179	19555.58	8.58	other
SmUSP25	958	483	160	17442.09	6.18	other
SmUSP26	614	408	135	15323.64	6.14	other
SmUSP27	1388	594	197	21771.16	6.33	mitochondrion
SmUSP28	819	654	217	23668.86	9.93	mitochondrion
SmUSP29	830	531	176	17813.38	9.89	chloroplast
SmUSP30	2263	1134	377	40372.12	11.16	other
SmUSP31	1709	249	82	9019.26	8.01	other
SmUSP32	2021	627	208	23136.63	8.27	other
