# Supplementary material for: Functional Characterization of Selected Universal Stress Protein from Salvia miltiorrhiza (SmUSP) in Escherichia coli
Source: Genes (Basel). 2017 Sep 8;8(9):224. doi: 10.3390/genes8090224 (PMC5615357; doi:10.3390/genes8090224)
Supplement: Supplementary file 1 [file genes-08-00224-s001.zip › Table S4.pdf]

Table S4: Promoter sequences analysis of 32 SmUSP

Gene	ABRE	HSE	MBS
SmUSP1	3	0	1
SmUSP2	7	2	3
SmUSP3	3	2	1
SmUSP4	3	1	0
SmUSP5	0	3	1
SmUSP6	0	1	0
SmUSP7	0	1	2
SmUSP8	0	0	0
SmUSP9	0	1	1
SmUSP10	0	0	0
SmUSP11	3	2	3
SmUSP12	1	1	1
SmUSP13	3	1	1
SmUSP14	2	0	1
SmUSP15	0	0	0
SmUSP16	6	4	2
SmUSP17	0	2	1
SmUSP18	0	2	0
SmUSP19	3	1	1
SmUSP20	3	0	8
SmUSP21	3	0	2
SmUSP22	1	1	1
SmUSP23	4	0	0
SmUSP24	0	1	2
SmUSP25	0	0	0
SmUSP26	0	1	5
SmUSP27	0	0	1
SmUSP28	0	3	0
SmUSP29	2	1	1
SmUSP30	2	2	3
SmUSP31	1	0	0
SmUSP32	1	0	2

*Promoter sequences of *SmUSP15*, *SmUSP25*, *SmUSP8* contain 3,1,1 TC-rich repeats elements involved in defense and stress responsiveness respectively; *SmUSP25* contains 2 LTR elements involved in low-temperature responsiveness and *SmUSP11*, *SmUSP21* contains 1 circadian element involved in circadian control respectively.
