# Supplementary figures and images for: Functional Characterization of Selected Universal Stress Protein from Salvia miltiorrhiza (SmUSP) in Escherichia coli
Source: Genes (Basel). 2017 Sep 8;8(9):224. doi: 10.3390/genes8090224 (PMC5615357; doi:10.3390/genes8090224)

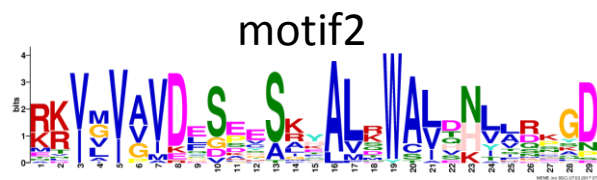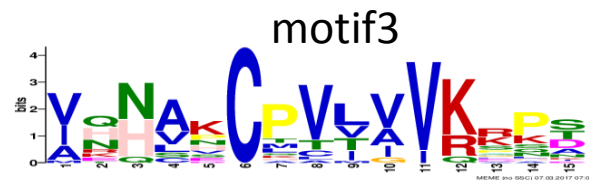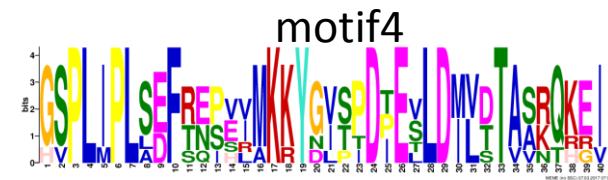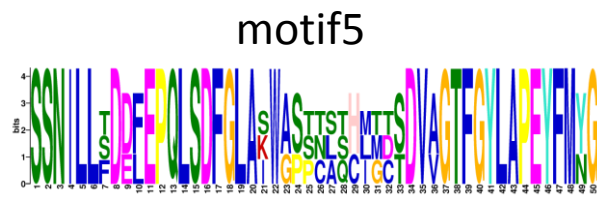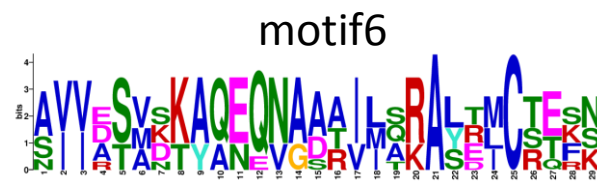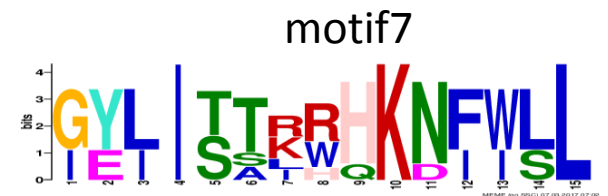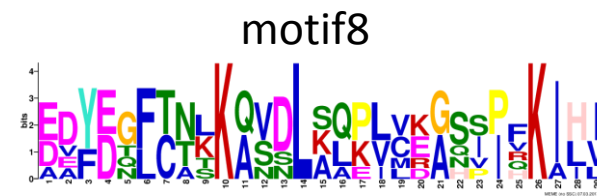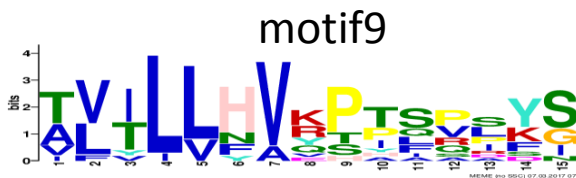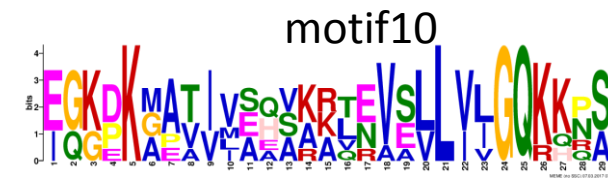

Figure S1: Motif 2-10 of SmUSP proteins

Supplement: Supplementary file 1 [file genes-08-00224-s001.zip › Figure S1.pdf]
